# PML/RARa inhibits PTEN expression in hematopoietic cells by competing with PU.1 transcriptional activity

**DOI:** 10.18632/oncotarget.11964

**Published:** 2016-09-10

**Authors:** Nélida Inés Noguera, Maria Liliana Piredda, Riccardo Taulli, Gianfranco Catalano, Giulia Angelini, Girish Gaur, Clara Nervi, Maria Teresa Voso, Andrea Lunardi, Pier Paolo Pandolfi, Francesco Lo-Coco

**Affiliations:** ^1^ Department of Biomedicine and Prevention, University of Rome “Tor Vergata”, Rome, Italy; ^2^ Neuro-Oncohematology Unit, Santa Lucia Foundation, Rome, Italy; ^3^ Cancer Research Institute, Beth Israel Deaconess Cancer Center, Department of Medicine and Pathology, Beth Israel Deaconess Medical Center, Harvard Medical School, Boston, MA, USA; ^4^ Department of Medical and Surgical Sciences and Biotechnologies, University of Roma “La Sapienza”, Rome, Italy; ^5^ Centre for Integrative Biology (CIBIO), University of Trento, Trento, Italy

**Keywords:** PTEN, PML-RARA, PU.1, oncosuppressor

## Abstract

Acute promyelocitic leukemia (APL) is characterized by the pathognomonic presence in leukemic blasts of the hybrid protein PML/RARA, that acts as a transcriptional repressor impairing the expression of genes that are critical to myeloid differentiation. Here, we show that primary blasts from APL patients express lower levels of the oncosuppressor protein PTEN, as compared to blast cells from other AML subtypes or normal bone marrow, and demonstrate that PML-RARA directly inhibits PTEN expression. We show that All-*Trans* Retinoic Acid (ATRA) triggers in APL cells an active chromatin status at the core regulatory region of the PTEN promoter, that allows the binding of the myeloid-regulating transcription factor PU.1, and, in turn, the transcriptional induction of PTEN. ATRA, via PML/RARA degradation, also promotes PTEN nuclear re-localization and decreases expression of the PTEN target Aurora A kinase. In conclusion, our findings support the notion that PTEN is one of the primary targets of PML/RARA in APL

## INTRODUCTION

Acute Promyelocytic Leukemia (APL) is characterized by the presence of the hybrid protein Promyelocytic Leukemia / Retinoic Acid Receptor Alpha (PML-RARA) originated by the t(15;17) balanced chromosomal translocation. The chimeric protein has pleiotropic effects and induces APL disease onset and progression [1, 2], by impairing the formation of functional PML nuclear bodies (PML NBs) [3], downregulating tumor suppressor genes [4, 5] through the recruitment of chromatin remodeling enzymes (e.g. co-repressor proteins and histone deacetylases) on their promoters [6], and deregulating transcriptional factors that are critical for myeloid differentiation [6–8]. The precise leukemogenic mechanism of the PML/RARA oncoprotein has not been fully elucidated, and its presence in transgenic mice is not *per se* sufficient to cause leukemia, but leads to a myeloproliferative syndrome [6–9]. Despite this, leukemic cells are addicted to *PML/RARA* and its degradation is associated with complete remission of the disease [10].

Peculiar to APL is the exquisite sensitivity to all-trans retinoic acid (ATRA) treatment. Pharmacological doses of ATRA are capable of reverting the leukemic phenotype, enabling terminal differentiation of promyelocytic blasts and disease remission [11–14]. ATRA is thought to act not only by antagonizing PML-RARA-dependent gene regulation, thereby favoring terminal differentiation, but also by degrading the hybrid protein in leukemia initiating cells (LICs) [14, 15].

In the myeloid compartment, PU.1, an ETS transcription factor known to regulate myeloid differentiation, has a well-established role in leukemia suppression [16, 17]. Silencing of PU.1 in the adult hematopoietic tissue produces dysfunctional stem cells and impairs granulopoiesis, by inducing a maturation block [18]. Furthermore, its inactivation causes myeloid leukemia in rats [19] and in irradiated mice [20, 21]. Interestingly, PU.1 interacts with both RARA and PML/RARA, and while PML/RARA has been demonstrated to downregulate PU.1 [22], ATRA treatment restores PU.1 levels [22], thus suggesting a tumor suppressive role of PU.1 in APL, through the transcriptional regulation of specific downstream target genes.

Phosphatase and Tensin homologue deleted on chromosome 10 (PTEN) is a protein and lipid phosphatase [23] with potent oncosuppressive functions, which is frequently inactivated in solid tumors [23]. Interestingly, PTEN also plays a pivotal role in the self-renewal of hematopoietic stem cells since its ablation has been demonstrated to promote exhaustion of normal hematopoietic stem cells (HSCs), and generation of leukemia-initiating cells (LICs) [24, 25]. Unexpectedly, however, only rare PTEN somatic mutations or genetic deletions have been reported in patients with acute myeloid leukemia (AML), including APL [26]. Notably, the study of hypomorphic PTEN mouse models, demonstrates that even subtle downregulation of PTEN levels can increase cancer susceptibility and favor tumor progression in specific tissues [27].

Here, we show that primary APL blasts are characterized by significantly lower levels of PTEN compared to blasts from other AML subtypes or normal bone marrow. We demonstrate that PML/RARA is directly involved in the downregulation of PTEN expression, while ATRA-treatment increases PTEN levels by inducing PU.1 transcriptional activity.

Our findings unveil a novel essential oncogenic activity of PML/RARA and further decrypt the mechanistic explanation of ATRA efficacy in eradicating t(15;17) APL.

## RESULTS

### PTEN expression is downregulated in APL compared to other AML subtypes

**Figure 1:**
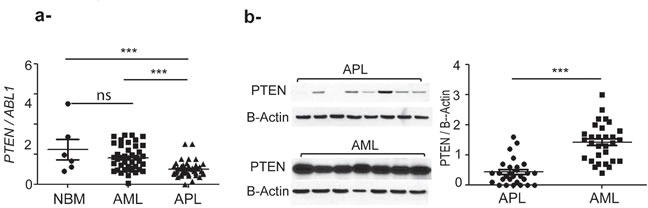
PTEN expression is significantly higher in primary AML compared to APL and normal bone marrow (NBM) samples **a***. PTEN* mRNA expression level, ns: no significative by unpaired t test. ***: p<0.0005 by unpaired t test. **b**. PTEN protein expression level. ***: p<0.0005. The nonparametric Mann–Whitney U test was used to compare continuous variables according to different groups.

*PTEN* mRNA level was measured using quantitative RT-PCR in 42 APL patients (mean value 1.5 ± 0.8), and compared to that of 42 AML patients (mean value 2.6±1.2) and 6 normal bone marrow (NBM) (mean 3.5±2.5) (Figure 1a). AML samples were characterized by heterogeneous PTEN expression, which was significantly higher than that of APL (p< 0.0001). PTEN protein levels in APL (n=28 samples, mean + SEM: 0.42 ± 0.42) were also significantly lower than that of other AML (n=30 samples, mean + SEM: 1.4 ± 0.6) (p<0.0001) (Figure 1b).

### ATRA induces PTEN expression

**Figure 2:**
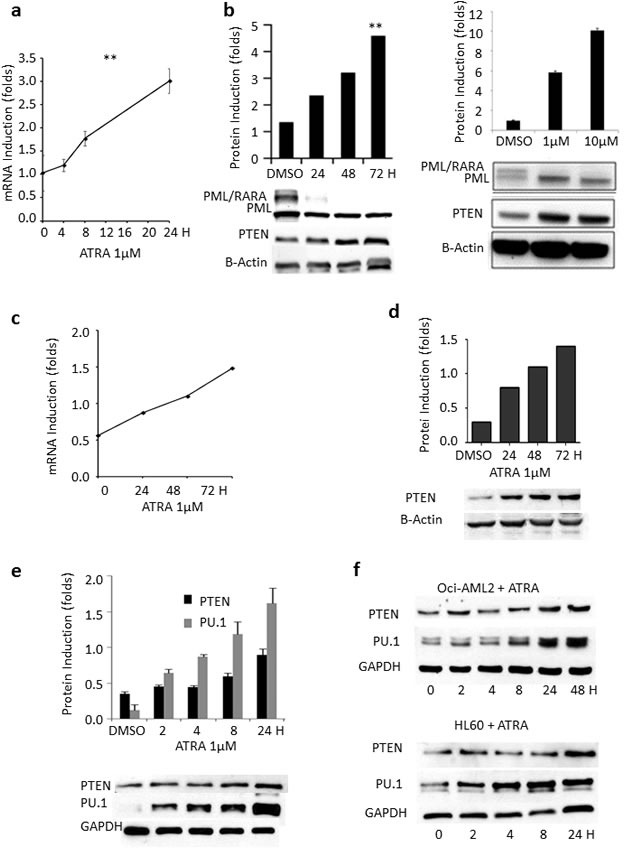

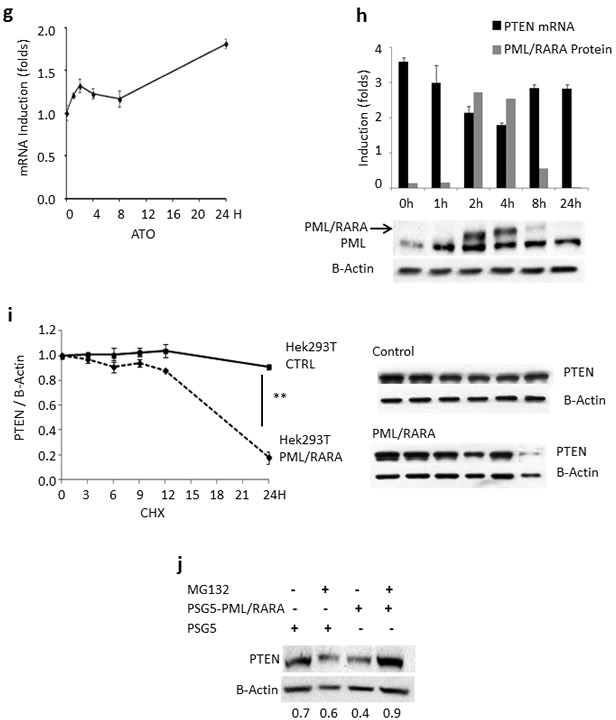
Effect of ATRA, ATO and PML/RARA on PTEN expression **a**. *PTEN* mRNA levels steadily increase in lysates from NB4 cells treated with ATRA, compared to DMSO. **: p<0.005 by unpaired t test. **b**. PTEN protein levels steadily increase in a time- and dose dependent manner in lysates from NB4 cells treated with ATRA, compared to DMSO. **: p<0.005 by unpaired t test. **c**. Induction of *PTEN* mRNA by ATRA in primary APL samples. **d**. PTEN protein is induced by ATRA in primary APL samples. **e**. Upon ATRA treatment, there is a parallel increase of PU.1 and PTEN protein expression. **f**. PTEN and PU.1 protein levels steadily increase upon ATRA treatment in lysates from OCI-AML2 and HL60 cells. **g***. ATO induces PTEN* mRNA expression in lysates from NB4 cells. **h**. PML/RARA protein is induced at 2 hours in PR9 cells treated with ZnSO4 and results in a decrease of *PTEN* mRNA. The position of the PML/RARA protein is indicated by the arrowhead. **i**. PML/RARA reduces PTEN half-life. HEK293T cells transfected with GFP-PTEN and PSG5-PML/RARA constructs or GFP-PTEN and (PSG5) control plasmid were treated with cycloheximide (CHX) (100μg/ml) over 24 hours. PML/RARA enhanced PTEN degradation at 24 hours. **: p<0.005 by unpaired t test. **j**. Proteasome inhibition rescues PTEN expression. The addition of the proteasome inhibitor MG132 to HEK293T cells transfected with the GFP-PTEN and PSG5-PML/RARA constructs or with GFP-PTEN and PSG5 control plasmid rescues PTEN from PML/RARA-induced degradation.

To study possible mechanisms dampening PTEN levels in APL, we took advantage of a panel of PML/RARA-positive and –negative cell lines. Following *in vitro* treatment of the PML/RARA positive NB4 cells with ATRA, *PTEN* mRNA and protein expression stably increased in a time-and dose-dependent manner (Figure 2a-2b). Dimethyl sulfoxide (DMSO) was used as a control. Similarly, *ex vivo* experiments on primary blasts from two APL patients showed that *PTEN* mRNA and protein expression levels increased over time during ATRA treatment, up to 2.5 and 4.6 folds at 72 hours, respectively (Figure 2c-2d).

Interestingly, up-regulation of PU.1 levels upon ATRA treatment has been described by Gu and colleagues in U937 [28], a PML/RARA negative histiocytic lymphoma cell line. Accordingly, treatment with ATRA promoted upregulation of PU.1 (and PTEN) both in PML/RARA-positive NB4 cells (Figure 2e) and in the PML/RARA-negative OCI-AML2 and HL-60 AML cell lines (Figure 2f), definitely suggesting the existence of PML/RARA-dependent and -independent mechanisms by which ATRA can induce PU.1 transcription.

### PML/RARA and PTEN expression are inversely correlated

In order to establish whether the effect of ATRA on PTEN was due to direct induction rather than to PML/RARA deprivation, we treated NB4 cells with 1μM Arsenic Trioxide (ATO), which is known to be able to degrade the PML/RARA protein without interfering with PTEN promoter. We observed about 60% enhancement of PTEN mRNA expression during the first 24 hours (Figure 2g).

To further investigate the possible connection between PTEN transcriptional regulation and PML/RARA expression, we used PR9 cells, a cell line expressing PML/RARA through a Zinc-inducible promoter. 100 μM ZnSO_4_ induced expression of PML/RARA at 2 to 4 hours, concomitant with a significant decrease of *PTEN* mRNA (Figure 2h). PML/RARA however dropped to very low levels 8 hours after Zinc induction, while *PTEN* transcript levels almost fully normalized (Figure 2h), strongly supporting the role for PML/RARA in *PTEN* transcriptional regulation.

To investigate whether PTEN regulation by PML/RARA occurred also at the post-translational level, we measured the protein life span in the presence or absence of the hybrid transcript. We co-transfected kidney epithelial HEK293T cancer cells with GFP-PTEN and PSG5-PML/RARA constructs or with GFP-PTEN and PSG5 control plasmid. Treatment of these cells with cycloheximide inhibited protein neo-synthesis at time zero. In the presence of PML/RARA, PTEN half-life decreased at 24 hours compared to control (0.91±0.02 vs 0.18±0.07, p = 0.004, Figure 2i). To further investigate the mechanism of PTEN degradation by PML/RARA, we then treated HEK293T cells with the proteasome inhibitor MG132, 24 hours after transfection with GFP-PTEN and PSG5-PML/RARA constructs, or with GFP-PTEN and PSG5 control plasmid. GFP-PTEN protein was degraded in the presence of PML/RARA while its level was stable in the control. Inhibition of proteasome function by cycloheximide rescued GFP-PTEN from PML/RARA-induced degradation (Figure 2j).

Overall, these data demonstrate that PML/RARA impairs PTEN tumor suppressive functions by promoting the transcriptional repression of the *PTEN* gene and also increasing its degradation. This is fully coherent with the fact that PML-RARA can also interfere with PML function and its ability to oppose HAUSP, hence causing PTEN nuclear exclusion [5] and its rapid turnover.

### PU.1 strongly binds to the PTEN promoter after PML/RARA degradation, and promotes chromatin activation in the PTEN core promoter region

**Figure 3:**
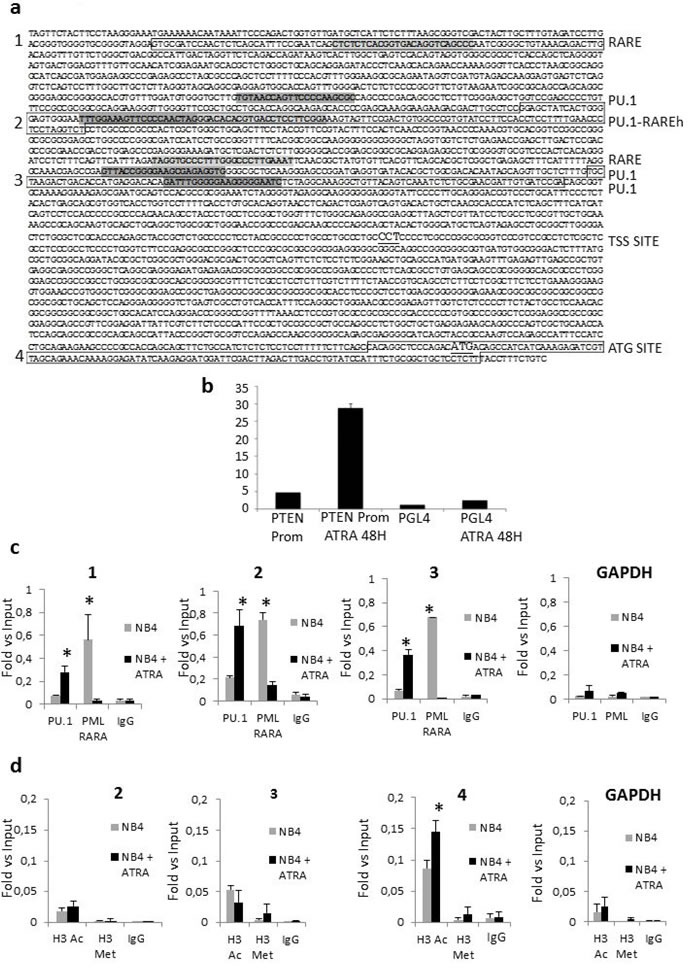
Regulation of PTEN expression **a**. The sequence of the promoter region of PTEN with indication of putative RARE and PU.1 binding sites is shown. Canonical retinoic acid responsive elements (RAREs) comprise direct repeats (DR) of two [A/G]G[G/T]TCA [RARE half (RAREh)], separated by five (DR5) or two (DR2) random nucleotides. Sequences in boxes 1, 2, 3 and 4 represent amplified regions. The TSS site is underline. The ATG site is underlined. **b**. Luciferase expression of NB4 cells electrophorated with either PGL4-PTEN promoter or PGL4 backbone construct, after 48hours of ATRA treatment. **c**. ATRA treatment enhances binding of PU.1 to the PTEN promoter region, while binding of PML/RARA decreases. The degree of PU.1and PML/RARA binding to DNA was measured in NB4 cells by a quantitative ChIP assay after treatment with ATRA. Data are shown as fold-enrichment of ChIP DNA versus input DNA. GAPDH was used as a negative control. Graphics 1, 2, and 3 match the amplified regions boxed in figure 3a. All data shown in the figure are representative of at least three independent experiments. *: p<0.05 by unpaired t test. **d**. Modifications of the histone markers, H3K14Ac and H3K27Me3, at the PTEN promoter region, by ChIP assays. Quantitative ChIP assays were performed to examine the acetylation and methylation of histone, H3K14Ac (white bars) and H3K27Me3 (black bars), at the promoter region of PTEN in NB4 cells after treatment with ATRA. The bars represent the relative levels of the q-PCR product of PTEN promoter associated with these specific histone modifications under the indicated conditions after immunoprecipitation with histone-specific antibodies, normalized to the H3 total level. Data are shown as fold enrichment of ChIP DNA versus input DNA. The bars represent the mean ± SD. GAPDH was used as negative control. Graphics 2,3 and 4 match the amplified regions boxed in figure 3a. All data shown in the figure are representative of at least three independent experiments. *: p<0.05 by unpaired t test.

We analyzed the PTEN promoter region for putative regulatory sequences and identified two RARE, three PU.1, and one PU.1/RARE half motifs (Figure 3a). Interestingly, ATRA is known to strongly induce PU.1 expression in APL cells, leading to neutrophil differentiation [22].

In order to mechanistically define the possible role of PML/RARA and PU.1 in *PTEN* gene transcription, we transfected NB4 cells using a PTEN promoter luciferase reporter construct, containing the 2Kb regulatory sequence upstream the ATG. ATRA treatment resulted in a strong activation of luciferase at 48 hours (Figure 3b), indicating the relevance of these regulatory motifs for the expression of PTEN. Then, we performed a ChIP assay on NB4 cells to analyze binding of PML/RARA and PU.1 to the putative regulatory sequences of the *PTEN* promoter (Figure 3c). GAPDH amplification was used as a negative control in all experiments. As shown in Figure 3c, ATRA increased PU.1 binding to the PTEN promoter region, in particular to the PU.1/RARE half motif (−2207 to −2163), 24 hours after treatment. At the same time, the amount of PML/RARA bound to these regions significantly decreased (Figure 3c).

It is known that PML/RARA inhibits PTEN gene expression in NB4 cells mainly *via* histone de-acetylation. The ChIP assay showed that ATRA treatment for 24 hours increased the acetylation status of lysine 14 on histone 3 (H3K14ac), a mark of transcriptionally active chromatin, in the PTEN promoter region surrounding the ATG transcription initiation site (Figure 3a and 3d). On the contrary, no changes in the transcriptional repressive mark H3K27me3 were detected in the same region (Figure 3d).

These results indicate that reduction of PML/RARA in APL due to ATRA treatment [29] unlocks *PTEN* and *PU.1* promoters, inducing an increase in *PU.1* and *PTEN* expression.

### ATRA treatment of NB4 cells restores PTEN nuclear localization and function

**Figure 4:**
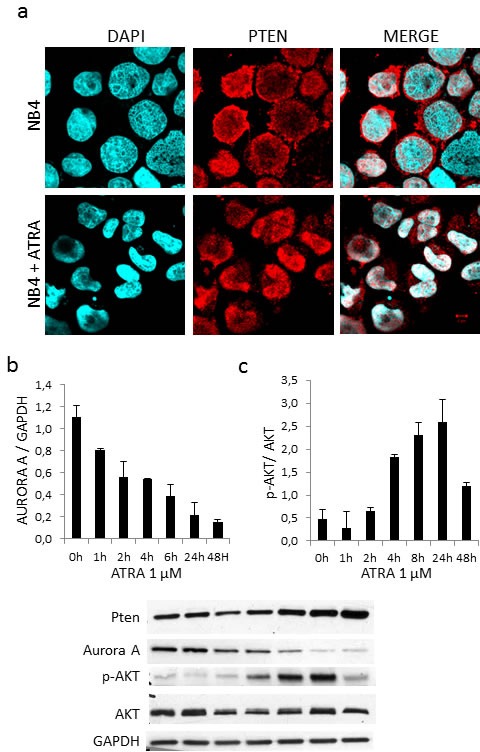
ATRA treatment restores PTEN localization and function **a**. Microscopy analysis of PTEN localization in NB4 cells treated with 1mM ATRA for 48 hours **b**. Time course treatment of NB4 cells with 1mM ATRA and analysis of Aurora A, p-AKT and PTEN expression levels.

We previously demonstrated that Aurora A (serine/threonine protein kinase 6), which is part of the machinery controlling cell cycle progression, is regulated by PTEN *via* APC/C activation, APC-CDH1 complex formation and consequent ubiquitination and degradation [30]. As we previously reported [3], treatment of NB4 cells with 1μM ATRA for 48 hours induced progressive PTEN protein accumulation and nuclear re-localization (Figure 4a). Importantly, protein quantification on Western blot showed a continuous decrement of Aurora A protein to a maximum of three folds at 48 hours (Figure 4b), thus proving that also in APL nuclear PTEN triggers APC/CDH1-dependent Aurora A degradation.

PTEN is known to negatively regulate pAKT levels, which are low in APL and APL cell lines as NB4. ATRA treatment of NB4 cells induced PTEN expression together with AKT phosphorylation, (Figure 4c).

## DISCUSSION

PU.1 downregulation or suppression causes AML [19–21], suggesting its pivotal role as tumor suppressor gene also in acute leukemia. Recent insights disclose a role of PU.1 in HSCs, as regulator of cell cycle, by limiting HSC proliferation and preserving HSC functional integrity. A positive auto-regulatory loop has been described in HSCs of adult mice to guarantee adequate PU.1 expression [31]. Similar to PTEN [32], PU.1 mediates its functions *via* gradual fluctuations in its expression levels. A tight regulation of PU.1 expression is critical for cell fate determination in the endothelial and hematopoietic compartments and in the hematopoietic lineages. It is expressed at low levels in uncommitted stem cells and prevents excessive proliferation, as levels increase, there is loss of proliferative potential and commitment, with higher levels promoting hematopoietic over endothelial fate. During B-cell development, PU.1 level decreases, whereas it increases during macrophage development [33–35]. In myeloid leukemic cells, PU.1 overexpression induces differentiation [22]. In particular, restoring sufficient levels of PU.1 functional protein can partially overcome the maturation arrest mediated by the PML/RARA and PLZF/RARA fusion proteins [36]. Of note, PU.1 overexpression does not *per se* interfere with LICs and does not cure the disease [17]. Factors that induce transcriptional activation in PML/RARA-transformed cells suffice just to initiate differentiation, whereas only a profound and long-lasting deprivation of PML/RARA function causes the loss of clonogenic activity, dissociating simple maturation of still leukemogenic blasts from true eradication of LICs [14].

**Figure 5:**
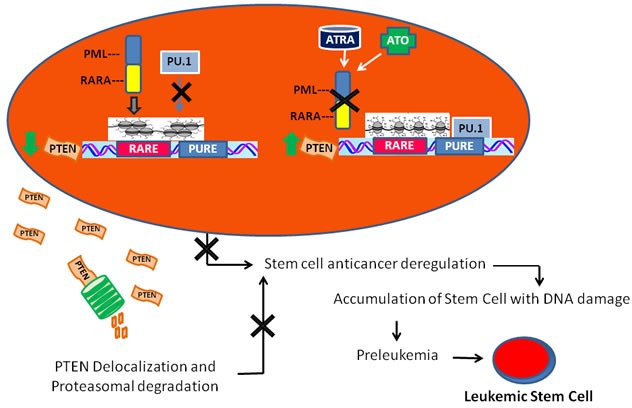
A model of leukemogenesis induced by PTEN deregulation PML/RARA inhibits the transcription of RA target genes, thus blocking myeloid differentiation, and prevents PU.1 binding to the PTEN promoter region. This causes a major decrease of PTEN production that, together with reduced PTEN lifespan due to its ubiquitination and cytoplasmatic localization, highly contributes to compromise its anti-oncogenic activity. In time, this may lead to the development of leukemia initiating progenitors.

We show here that PTEN expression is directly suppressed by PML/RARA, which binds to PTEN promoter, and that degradation of the hybrid protein by ATRA or arsenic trioxide promotes *PTEN* expression in cells harboring the t(15;17) translocation. Actually ATRA-treatment, restores an active chromatin status at the *PTEN* core promoter region, and induces *PTEN* transcription by inducing PU.1 expression and its recruitment to specific recognition motifs in the *PTEN* promoter region. Importantly, ATRA-treatment re-localizes PTEN in the nucleus of differentiating cells and this impacts on the abundance of the APC-CDH1-target Aurora A. Therefore, as depicted by our model (Figure 5), the *PTEN* promoter region is repressed in APL by the PML/RARA repressor complex, while residual amount of PTEN is exported from the nucleus to the cytoplasm, and degraded. In this scenario, the lack of PTEN anticancer control could play a pivotal role by favoring cellular proliferation and survival, in turn allowing the accumulation of additional genetic events towards leukemogenesis, as suggested by the latency between expression of the hybrid protein and leukemia onset in PML/RARA transgenic mice [6, 9].

The mechanisms of ATRA-induced differentiation are various: among others, it directly induces PU-1 [22] and PTEN expression, beyond removing PML/RARA blockage. This is in line with our finding that ATRA induces PU.1 and PTEN expression in different AML cell lines regardless of the presence of PML/RARA. This may also suggest that PTEN and PU.1 upregulation could be relevant to the antitumoral effects of ATRA in other AML subtypes, beyond APL.

## MATERIALS AND METHODS

### Primary patient samples

Bone marrow samples were collected at diagnosis from AML and APL patients, treated at the Department of Hematology of the University Tor Vergata of Rome. Infiltration of bone marrow by leukemic blasts was >70% in all patients included in the study. APL diagnosis was confirmed in all cases by detection of the disease-specific *PML-RARA* fusion gene. Written informed consent to the study was obtained from all patients, and the study has been approved by the IRB of Policlinico Tor Vergata, Rome.

### Cell line culture and treatment

The human cell lines Oci-AML2, HL60, the APL-derived cell line NB4 carrying the t(15;17) translocation and expressing the PML/RARA fusion protein, and PR9 (a zinc-inducible PML/RARA model constructed from the U937 cell line) [4] were grown at 37°C in a humidified atmosphere of 5% CO_2_ in air, in RPMI medium (GIBCO-BRL, Grand Island, NY, USA), supplemented with 10% fetal bovine serum (FBS) (GIBCO-BRL, Grand Island, NY, USA), 20 mM Hepes, 100 g/mL penicillin and 100 g/mL streptomycin (GIBCO-BRL, Grand Island, NY, USA). These cell lines were kindly provided by Emanuela Colombo, European Institute of Oncology, Milan. ATRA, ATO and ZnSO4 (all from Sigma-Aldrich, Steinheim, Germany), were respectively dissolved in DMSO (10 mM stock solution, SS), and sterile water (1 MSS).

### Western blot analysis

Cell pellets were re-suspended in lysis buffer containing 10 mMTris–HCl (pH 7.4), 5 mM EDTA, 150 mMNaCl, 1% Triton X-100, 250 μM orthovanadate, 20 mMβ-glycerophosphate and protease inhibitors (Sigma-Aldrich, Steinheim, Germany). Lysates were centrifuged at 10,000 g for 15 minutes at 4°C and supernatants were stored at −80°C. Protein concentration was measured by the Bradford Assay (#500-0006; Bio-Rad, München, Germany). Thirty microgram aliquots of proteins were re-suspended in a reducing Laemmli Buffer (with β-mercaptoethanol), loaded onto a 10% polyacrylamide gel, and then transferred to nitrocellulose membranes. After blocking with 5% milk (Fluka, Sigma-Aldrich, Saint Louis, USA), the membranes were incubated with specific antibodies. Horseradish peroxidase-conjugated IgG preparations were used as secondary antibodies, and the immunoreactivity was determined by the enhanced chemiluminescence (ECL) method (Amersham, Buckinghamshire, UK). The autoradiograms were scanned and exported for densitometry analysis. Protein signal intensities were measured using the Quantity One Software (Bio-Rad Laboratories, Hercules, CA, USA). Signal quantity was normalized using the unrelated proteins β-actin (Cell Signaling Technology, Beverley, MA, USA), or GAPDH (Abcam plc, Cambridge, UK).

### Quantitative real-time expression analysis

Leukemic blasts were obtained after Ficoll-Hypaque centrifugation of bone marrow mononuclear cells. Total RNA was extracted using Trizol (Life Technologies, Invitrogen, USA). For cell lines, 2 × 10^6^ cells were collected as starting material for RNA extraction. Reverse transcription was performed on 1 μg total RNA using a standardized protocol (Applied Biosystems, Foster City, USA). The primers were obtained from IDT (San Jose, California, USA). Primer sequences were ABL-foward5′-TGGAGATAACACTCTAAGCATAACTAAAGGT-3′, ABL-reverse: 5′-GATGTAGTTGCTTGGGACCCA-3′ and ABL-probe 5′-FAM-CCATTTTTGGTTTGGGCTTCACACCATT-BHQ-3′; PTEN-foward5′-GCTCTATACTGCAAATGCTATCG-3′; PTEN-reverse5′-CCACAAACAGAACAAGATGCT-3′; PTEN-probe: 5′-FAM/TCTTCATAC/ZEN/CAGGACCAGAGGAAACCT/3IABkFQ/-3′.

Expression levels of the genes of interest and of the control were quantitated using TaqMan® Universal PCR Master Mix (Applied Biosystems, Warrington, UK).

### Reporter plasmids and luciferase assay

The PTEN regulatory promoter region was subcloned in PGL4.1 FLuc reporter plasmid (Promega, Madison, USA), using the following primers: forward: 5′-CCCTCTTTAGACTTTGCTAGGC-3′ and reverse: 5′-GGTAGGAGGGGGCAGAGC-3′. NB4 cells were electroporated using the Amaxa Nucleofector system according to the manufacturer's protocol. Briefly, 2 × 10^6^ cells were co-transfected with 2 ug of PGL-4 PTEN promoter and 200 ng of pRLTK plasmid (Promega, Madison, USA) encoding for renilla luciferase gene as internal control. Electroporation was performed by re-suspending the cells in 100 μl Cell Line Nucleofector® Solution V, Program X-001. Immediately after electroporation, cells were treated with 1uM ATRA for 48hrs. Luciferase was measured using the Dual-Luciferase Reporter Assay System (Promega, Madison, USA), according to the manufacturer's instructions. Luminescence was measured with the “Dual Glow” protocol of the Glowmax MULTI + Detection System (Promega, Madison, USA).

### ChIP assay

ChIP assays were performed as previously described [37], with some modifications. Briefly, DNA was double-crosslinked to proteins with 1% formaldehyde (Sigma, St Louis, USA). After incubation for 10 minutes at RT, glycine was added to a final concentration of 0.125 M, for 5 minutes. The cells were washed twice with PBS 1 x, cell lysis buffer (10 mMTris pH 8.0, 100 mMNaCl and 0.2 % NP40) was added to the samples and incubated on ice for 30 minutes. Nuclei were pelleted by microfuge at 1500 rpm at 4°C, and after addition of the nuclear lysis buffer (50 mMTris 8.1, 10 mM EDTA and 1 % SDS) were incubated on ice for 30 minutes. Chromatin fragments of around 200–300 bp were obtained by sonication, using a Branson Sonifier 450 Analog Cell Disruptor (30″ON, 45″ OFF, for a total time of 10 minutes at output 2). For each immunoprecipitation, 3 mg of antibodies were conjugated to magnetic beads (G-protein magnetic Beads, Invitrogen, Dynal, Oslo). The following antibodies were used in the ChIP assays: anti-PU.1 (T-21, sc-352, Santa Cruz Biotechnology, Inc. Dallas, USA), anti PML (H-238, sc5621, Santa Cruz Biotechnology, Inc. Dallas, USA), anti-Actil-Histone H3 (Lys14), anti-trimethyl-Histone H3 (Lys27) (#06-599 and 07-449, Millipore, Darmstadt, Germany), anti-Histone H3 (ab1791, Abcam, Cambridge, UK) and normal rabbit IgG (#2729, Cell Signaling Inc. Massachusetts, USA). After extensive washing, bound DNA fragments were eluted and analyzed by quantitative PCR using the SYBR Green Master Mix (Applied Biosystems, Warrington, UK). ChIP signals were normalized against the input and expressed as relative enrichment of the material, precipitated by the indicated antibody binding to the PTEN promoter [relative quantification using the comparative Ct method (2-(Ct sample-Ct input)]. Chip primers are: P1F-5′-gtgcgatccaactctcagca-3′ P1R-5′-GCAAGTCTGTTTACAGCCCCG-3′, P2D-5′-ggagctatcactggggagtg-3′ P2R-5′- GAGGAGACCTAGGAGGGTTC-3′; P3F-5′-GCTAAGACTGACACCATGAGGAC-3′ P3R-5′-TCGGATCACAATCGTTCGCAG-3′ P4F-5′-CAGGCTCCCAGACATGACAG-3′; P4R-5′-CGAATCCATCCTCTTGATATCTC-3′. The GAPDH gene was used as a negative control; forward: 5′-GTATTCCCCCAGGTTTACAT-3′ and reverse 5′-TTCTGTCTTCCACTCACTC-3′.

### Immunofluorescence assays

Cytospins from the NB4 cell line were prepared using a cytocentrifuge. Cells fixed with 4% paraformaldehyde (PFA) were permeabilized in PBS containing 0.2% Nonidet P-40 and blocked in 5% BSA. Slides were incubated overnight with the primary antibody (PTEN, clone 6H2.1, Cascade Bioscience Inc., Winchester, MA, USA), washed twice in PBS and incubated for 2 h with the secondary antibodies: Invitrogen Alexa Fluor 555-labeled goat anti-mouse (diluted 1:400 with PBS+BSA 2%). After nuclear counterstaining with 4′,6-diamidino-2-phenylindole (DAPI), slides were visualized using an Olympus BX61 fluorescent microscope equipped with a CoolSNAP EZ camera (Photometrics, Tucson, AZ, USA).

### Transfection experiments

To evaluate the effect of PML/RARA on PTEN half life, Hek293T cells were transfected with pSG5-PML/RARA or pSG5 constructs. Western blot assays were performed following treatment with the inhibitor of protein biosynthesis Cycloeximide (CHX) over 24 hours.

### Statistical analysis and bioinformatics

All statistical analyses were conducted using the program GraphPad Prism5. Promoter sequences were retrieved from the National Center for Biotechnology Information online database (www.ncbi.nlm.nih.gov). Prediction of transcription factor binding sites was performed using the MatInspector (www.genomatix.de) and ALGGEN PROMO (http://alggen.lsi.upc.es) software.­­
